# Two Ektaphelenchinae Paramonov, 1964 (Nematoda: Rhabditida) from Iran have tripartite stylet, with similar observations in other species

**DOI:** 10.1371/journal.pone.0215731

**Published:** 2019-05-13

**Authors:** Majid Pedram

**Affiliations:** Department of Plant Pathology, Faculty of Agriculture, Tarbiat Modares University, Tehran, Iran; Tata Institute of Fundamental Research, INDIA

## Abstract

Two ektaphelenchid nematodes representing one new and one known species are illustrated and characterized using morphological and molecular data. *Ektaphelenchus kanzakii* n. sp. is mainly characterized by its tripartite stylet having a well visible wide lumen, encompassing a sclerotized and acute anterior part (the conus), a short and slightly tapering middle part (the conophore) that is equally sclerotized but clearly separate from the conus, and a long posterior part that is cylindrical and only weakly sclerotized (the shaft) without basal knobs or swellings. It is further characterized by 863.5 (772–926) μm long females having 23.8 (21.2–27.0) μm long total stylet, distinctly annulated cuticle, three lines in lateral field, vulva at 76.6 (75.3–80.0)%, no rectum, vestigial anus in some individuals, conical posterior body end (tail) with narrow ventrally bent tip, common males in population with two pairs of caudal papillae (the single precloacal papilla and the third caudal pair lacking), spicules with dorsally bent tip and conical tail with sharp or blunt tip. The new species is morphologically compared with close species having conical posterior body end and stylet lacking basal knobs or swellings. Iranian population of *Devibursaphelenchus teratospicularis*, the second studied species, is characterized by 679.5 (620–709) μm long females having 18.6 (17.5–20.0) μm long total stylet with similar structure to the previous species, subcylindrical body end with widely rounded tip, and rare males with typical spicules of this species and a pair of precloacal and a pair of caudal papillae. Molecular phylogenetic studies of the two recovered species using small and large subunit ribosomal DNA (SSU and LSU rDNA) partial sequences revealed they have close phylogenetic affinities with *Ektaphelenchus obtusus* in both reconstructed trees. However, species of both genera *Ektaphelenchus* and *Devibursaphelenchus* don’t form monophyletic groups in SSU and LSU trees. New observations on stylet structure of the two presently studied and some other ektaphelenchid species having available light microphotographs (LM) yielded on definition of a new term “conophore” for the middle part of the ektaphelenchid-type tripartite stylet.

## Introduction

Currently 28 species are placed under the genus *Ektaphelenchus* Fuchs, 1937 [1] (24 in Hunt [2], plus four recently described species: *E*. *taiwanensis* Gu, Wang & Chen, 2013 [3], *E*. *ibericus* Gu, Wang, Chen & Wang, 2013 [4], *E*. *berbericus* Alvani, Mahdikhani-Moghadam, Giblin-Davis & Pedram, 2016 [5] and *E*. *oleae* Miraeiz, Heydari, Adeldoost & Ye, 2017 [6], thereafter). The genus is known in Iran with two representatives (*E*. *oleae* and *E*. *berbericus*, both of which are described form Iran, and apparently are endemic to the country after currently available reports).

*Devibursaphelenchus* Kakuliya, 1967 [7], one of the rare aphelenchoidid genera, currently includes five nominal species [8, 9] and is known in Iran with only one representative, *D*. *kheirii* Aliramaji, Pourjam, Atighi, Karegar & Pedram, 2014 [9], the last species added to the genus. A history of taxonomic studies on ektaphelenchid species and genera occurring in Iran is given by Pedram [10].

Inspecting of several bark and wood samples of dead or dying *Pinus* sp. in city of Tehran collected on late 2016-early 2017, yielded three ektaphelenchids, one of which was recently described as *Cryptaphelenchus varicaudatus* Pedram, 2017 [10]. The two other species belonged to one new and a known species of the respective genera *Ektaphelenchus* and *Devibursaphelenchus* and are studied and illustrated in present paper.

## Material and methods

### Ethics statement

No specific or official permits were required for collecting the wood or bark samples studied herein. The samples were obtained from common debris and woody wastes in public areas, and this study did not involve any endangered or protected species in Iran, nor were the sites protected in any way.

### Sampling, nematode extraction, mounting and drawing

Several wood and bark samples were collected from different parks in the city of Tehran on the late 2016-early 2017. The samples were mostly collected from common woody wastes or trunks of dead trees to be removed. The tray method of Whitehead and Hemming [11] was used to extract nematodes from the barks and woods. Nematodes of interest were handpicked under a Nikon SMZ1000 stereomicroscope, heat-killed by adding boiling 4% formalin solution, transferred to anhydrous glycerine according to De Grisse [12], mounted on permanent slides, and examined using a Nikon Eclipse E600 light microscope. Photographs were taken using an Olympus DP72 digital camera attached to an Olympus BX51 microscope with differential interference contrast (DIC). Drawings were made using a drawing tube attached to the microscope and were redrawn using CorelDRAW software version 16.

### DNA extraction, PCR, sequencing

For extracting DNA, three females of the new species and two females of the studied population of *Devibursaphelenchus teratospicularis* Kakulia & Devdariani, 1965 [13] were hand-picked, individually examined on a temporary slide, photographed and transferred to a small drop of TE buffer (10 mM Tris-Cl, 0.5 mM EDTA; pH 9.0, QIAGEN Inc., Valencia CA, USA) on a clean slide and crushed using a cover slip. The suspension was collected by adding 20 μl TE buffer. The DNA samples (each in a separate tube) were stored at –20°C until used as PCR template. Primers for LSU rDNA D2-D3 amplification were forward primer D2A (5’ACAAGTACCGTGAGGGAAAGT 3’) and reverse primer D3B (5’TGCGAAGGAACCAGCTACTA3’) [14]. Primers for amplification of SSU rDNA were forward primer 988F (5´-CTCAAAGATTAAGCCATGC-3´) and reverse primer 1912R (5´-TTTACGGTCAGAACTAGGG-3´), forward primer 1813F (5´-CTGCGTGAGAGGTGAAAT-3´) and reverse primer 2646R (5´-GCTACCTTGTTACGACTTTT-3´) [15]. PCR was carried out in a total volume of 30 μl (19.2 μl distilled water, 3 μl 10x PCR buffer, 0.6 μl 10 mM dNTP mixture, 1.2 μl 50 mM MgCl_2_, 1.2 μl of each primer (10 pmol/μl), 0.6 μl of *Taq* DNA polymerase (5 unit/μl, CinnaGen, Tehran, Iran) and 3 μl of DNA template). The thermal cycling program for amplifying of both genomic fragments was as follows: denaturation at 95°C for 4 min, followed by 35 cycles of denaturation at 94°C for 30 s, annealing at 52°C for 40 s, and extension at 72°C for 80 s. A final extension was performed at 72°C for 10 min. The PCR products were sequenced in both directions using the same primers with an ABI 3730XL sequencer. Newly obtained sequences of the studied species were deposited into the GenBank database with accession numbers MH595681-MH595686 (for species and isolates names see trees).

### Phylogenetic analyses

The newly obtained sequences were compared with those of the other nematode species available in GenBank using the BLAST sequence similarity search program. For inferring phylogenetic relationships, two independent SSU and LSU datasets, as used by Pedram [10], were prepared. Each dataset was aligned using the Q-INS-i algorithm of online version of MAFFT version 7 (http://mafft.cbrc.jp/alignment/server/) (Katoh and Standley [16]). To eliminate the ambiguously aligned parts, the online version of Gblocks 0.91b [17] with all three less stringent parameters was used (http://molevol.cmima.csic.es/castresana/Gblocks_server.html). The model of base substitution was selected using MrModeltest 2 [18]. The Akaike-supported model, a general time reversible model, including among-site rate heterogeneity and estimates of invariant sites (GTR+G+I) was used in phylogenetic analyses of both SSU and LSU datasets. Bayesian analysis was performed using MrBayes v3.1.2 [19] running the chains for 5 million generations (for the both datasets). After discarding burn-in samples (the burn-in was set to 25%), the remaining samples were retained for further analyses. The Markov chain Monte Carlo (MCMC) method within a Bayesian framework was used to estimate the Bayesian posterior probabilities (BPP) of the phylogenetic trees [20] using the 50% majority rule. Tracer v1.5 software [21] was used to visualize the results of each run and check the effective sample size of each parameter. The output files of the phylogenetic programs were visualized using Dendroscope V.3.2.8 [22] and re-drawn in CorelDRAWsoftware version16. The BPP are given on appropriate clades.

### Nomenclatural acts

The electronic edition of this article conforms to the requirements of the amended International Code of Zoological Nomenclature (ICZN), and hence the new name contained herein is available under that Code from the electronic edition. This published work and the nomenclatural acts it contains have been registered in ZooBank, the online registration system for the ICZN. The ZooBank LSIDs (Life Science Identifiers) can be resolved and the associated information viewed through any standard web browser by appending the LSID to the prefix "http://zoobank.org/". The LSID for this publication is: urn:lsid:zoobank.org:pub:F6BD1382-59E4-466F-8DE3-D61AD22983FC. The electronic edition of this work was published in a journal with an ISSN, and has been archived and is available from the following digital repositories: PubMed Central, LOCKSS.

## Results

### *Ektaphelenchus kanzakii* Pedram n. sp.

urn:lsid:zoobank.org:act:78A8D73D-750B-4D6F-AF42-9E8472452685

### Measurements and morphology

**Fig 1:**
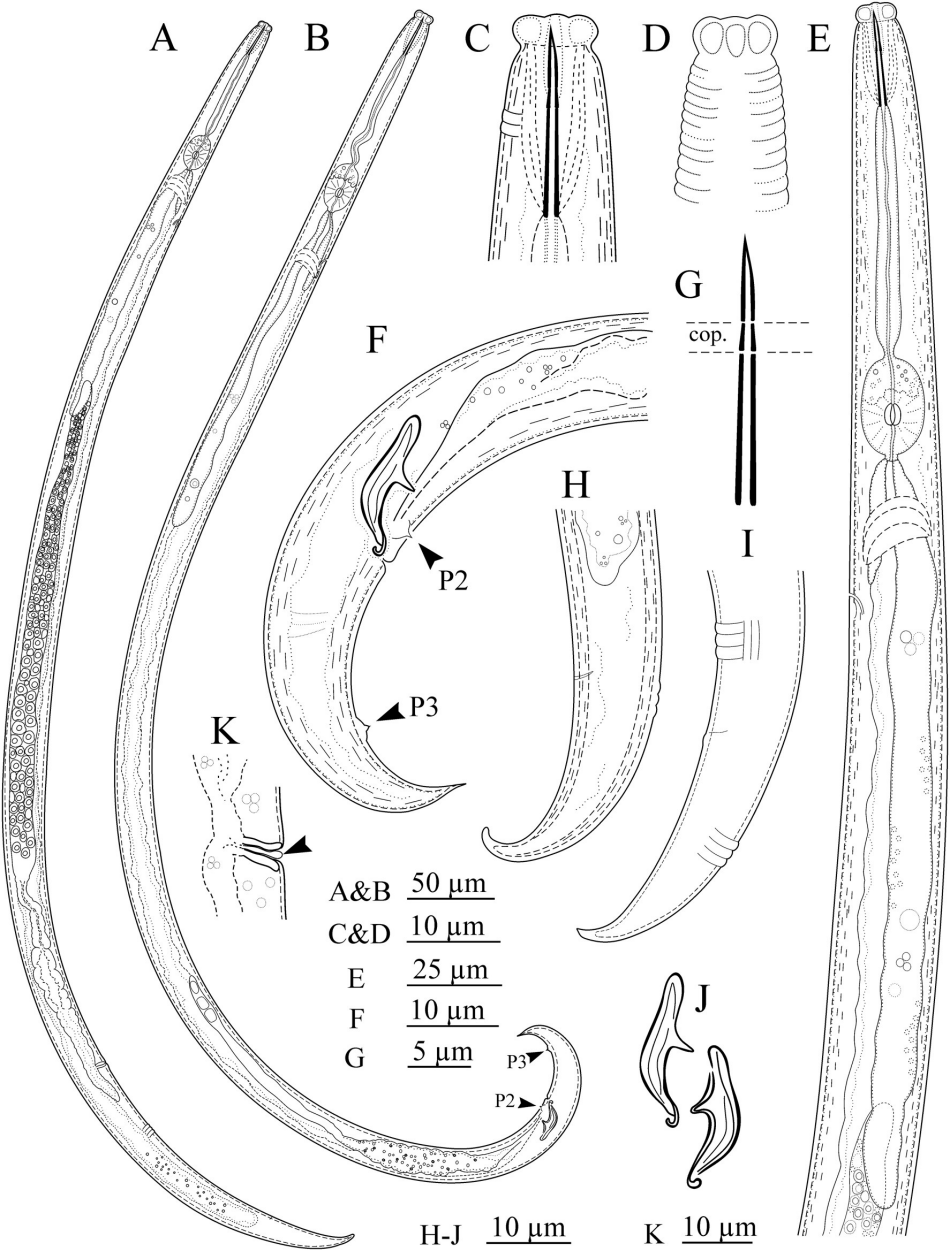
Line drawings of *Ektaphelenchus kanzakii* n. sp. A&B: Female and male entire body, C&D: Anterior end and corresponding details, E: Pharynx, F: Male posterior body region, G: Stylet structure (cop.: conophore), H&I: Female posterior body end, J: Spicules, K: Epiptygma-like small differentiation (arrowhead).

**Fig 2:**
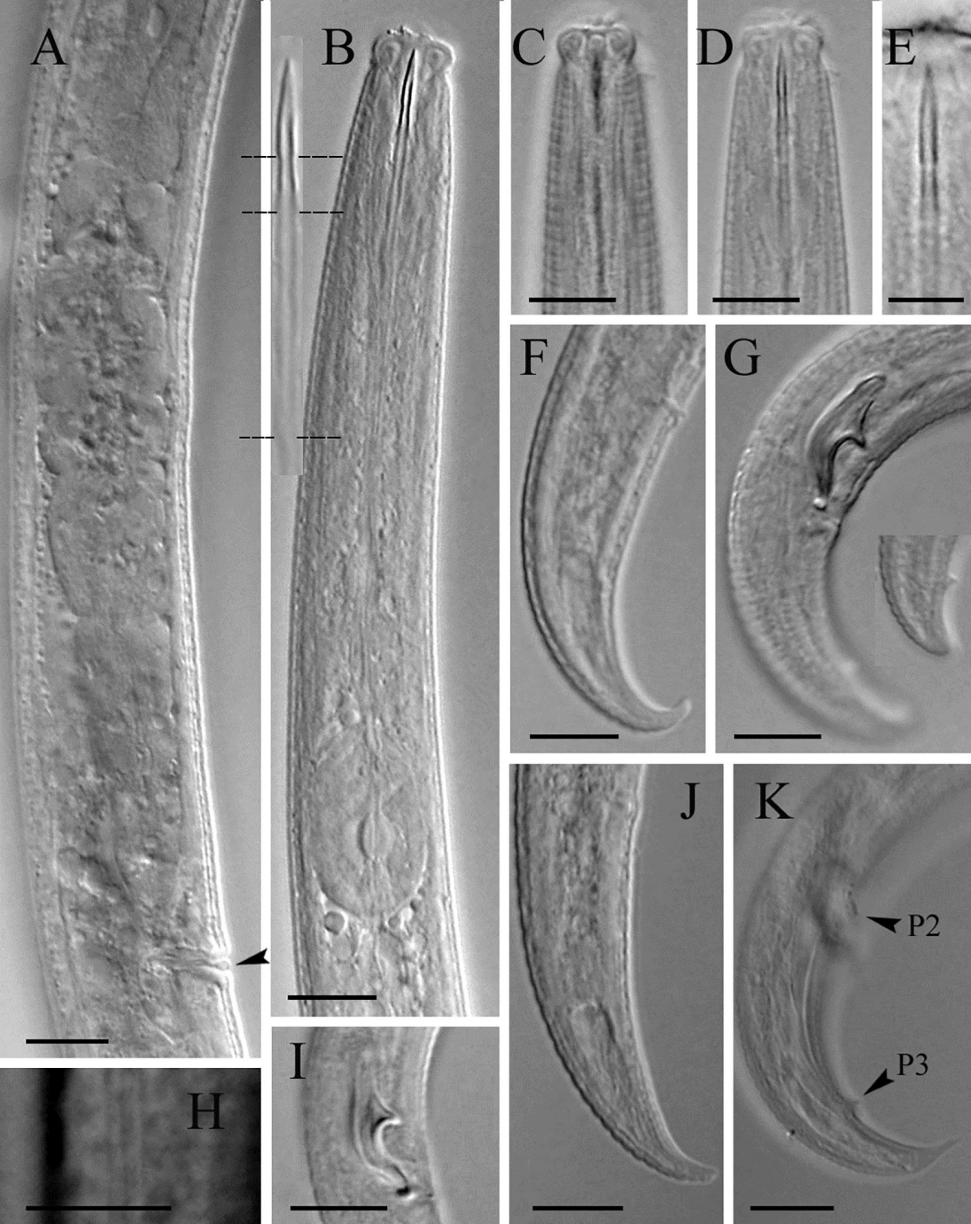
Light microphotographs of *Ektaphelenchus kanzakii* n. sp. A: Part of female reproductive system (epiptygma-like differentiation in vulva is visible; arrowhead), B: Part of pharynx (inset: Stylet in detail, ca. 20 μm long), C&D: Anterior end, E: Conus and middle part of stylet in high contrast, F&J: Female posterior body end, G&K: Male posterior body end, H: Lateral lines, I: Spicules. (All scale bars = 10 μm, except the inset in plate B, its conus 7 μm).

Morphometric data are described in Table 1 and morphological traits are shown in Figs 1 and 2.

**Table 1:** Morphometrics of *Ektaphelenchus kanzakii* n. sp. and Iranian population of *Devibursaphelenchus teratospicularis* Kakulia & Devdariani, 1965. All measurements are in μm and in the form mean±S.D. (range).

	*Ektaphelenchus kanzakii* n. sp.			*Devibursaphelenchus teratospicuaris*	
	Holotype	Paratypes		Iranian population	
n	Female	Females	Males	Females	Male
n	1	12	6	8	1
L	884	863.5±49.3	649.4±53.2	679.5±33.4	587
		(772–926)	(574–718)	(620–709)	
a	36.8	40.5±3.2	39.3±3.5	39.4±4.7	36.7
		(35.9–45.2)	(35.9–44.9)	(32.6–45.1)	
b	8.8	8.7±0.3	7.3±0.8	8.4±0.3	7.7
		(8.2–9.5)	(6.0–8.2)	(8.0–8.9)	
b'	3.3	3.3±0.3	2.8±0.3	2.9±0.2	2.8
		(2.8–3.5)	(2.4–3.0)	(2.6–3.0)	
c	-	-	18.7±2.2	-	19.6
			(16.3–21.9)		
c'	-	-	2.7±0.4	-	2.1
			(2.4–3.4)		
V	77	76.6±1.3	-	78.4±0.9	-
		(75.3–80.0)		(76.9–79.8)	
M^a^	25	26.3±2.0	27.5±1.3	26.0±1.8	26.3
		(24–31)	(26.2–29.7)	(24.5–28.5)	
Stylet-first part (conus)	6.5	6.2±0.3	5.8±0.2	4.8±0.3	5
		(6.0–6.8)	(5.5–6.0)	(4–5)	
Stylet-mid part (conophore)	3.5	3.3±0.4	2.9±0.2	3.5±0.4	3.5
		(2.5–3.7)	(2.5–3.5)	(2–4)	
Stylet-shaft	16	14.6±1.5	12.4±1.2	10.3±0.4	10.5
		(12–17)	(10.0–13.5)	(10–11)	
Total stylet length	26	23.8±1.8	21.1±1.4	18.6±1.0	19
		(21.2–27.0)	(18.5–22.5)	(17.5–20.0)	
MB%^b^	90	90.5±1.1	90.8±0.5	88.9±2.1	88
		(88.7–91.5)	(90.0–91.5)	(86.5–92.4)	
Nerve ring	114	108.8±4.3	97.0±7.5	94±6	89
		(104–116)	(90–105)	(85–100)	
E. pore	132	128.1±7.4	108.7±3.5	102.7±6.5	-
		(116–135)	(105–112)	(96–109)	
Hemizonid	135	129.2±5.8		108.0±2.3	-
		(118–136)		(106–111)	
Pharynx (anterior end to pharyngo-intestinal junction)	101	98.4±4.0	88.8±4.6	80.9±4.3	76
		(90–106)	(83–96)	(74–87)	
Head-vulva	681	661±38		532.0±25.5	-
		(587–705)		(493–556)	
Body width at median bulb level	19	17.2±1.4	14.2±0.4	14.4±0.5	-
		(15–19)	(14–15)	(14–15)	
at mid body	24	21.5±1.8	16.6±1.2	17.4±1.5	16
		(19–24)	(15–18)	(15.5–19.0)	
PUS	21.5	20.8±1.2		18.6±2.2	-
		(19–23)		(16.0–21.5)	
Cloaca or vestigial anus to distal body end (tail)	53	52.0±1.4	35.2±5.3	-	30
		(50–53)	(30–44)		

^a^: Stylet conus as percent to stylet total length

^b^: Anterior end to middle of metacarpal valve as percent to pharynx length

### Holotype

Adult female, mounted in pure glycerin and deposited in the nematode collection at Nematode Collection of the Faculty of Agriculture, Tarbiat Modares University, Tehran, Iran.

### Paratypes

Five paratype females and three paratype males deposited in the USDA Nematode Collection, Beltsville, MD, USA and six paratype females and three paratype males were deposited at WaNeCo collection, Wageningen, The Netherlands (http://www.waneco.eu/).

### Diagnosis

*Ektaphelenchus kanzakii* n. sp. is mainly characterized by its 863.5 (772–926) μm long females with distinctly annulated cuticle, offset cephalic region flat in anterior end, three lines in lateral field forming two equally distant lines, six equally sized separate lips, tripartite well developed stylet having wide lumen and simple base, well developed muscular metacorpus with sclerotized refractive valve plates, excretory pore far from metacorpus base, vulva at 76.6 (75.3–80.0)% with an epiptygma-like small differentiation, no rectum, vestigial anus in some individuals, conical posterior body end (tail) with narrow ventrally bent tip, common males in population with two pairs of precloacal and caudal papillae, spicules with dorsally bent distal tip and conical tail with sharp or blunt end.

### Etymology

This species is named in honor of my friend and colleague, Dr. Natsumi Kanzaki, an outstanding scientist in taxonomy of insect related nematodes.

### Type habitat and locality

The new species was recovered from bark samples of dead *Pinus* sp. having frass and galleries of bark beetles, collected in Chitgar forest park, city of Tehran, Tehran province, Iran, collected on late 2016-early 2017.

#### Description of taxa. Female

Body slender, slightly ventrally curved after fixation, gradually narrowing toward posterior end due to having a conical body end (tail). Cuticle distinctly annulated, annulus 2.1 (1.9–2.3) μm wide. Lateral field with three lines. Lip region anteriorly flat, separated from the rest of the body by a distinct constriction, 2.2 (2.1–2.5) times wider than tall, lips separated and equally sized. Stylet well developed, distinctly tripartite, having a well visible wide lumen, encompassing a sclerotized and acute anterior part (the conus), a short and slightly tapering middle part (the conophore) that is equally sclerotized but clearly separate from the conus, and a long posterior part ca. twice conus length long, that is cylindrical and only weakly sclerotized (the shaft) without basal knobs or swellings. The stylet aperture ca. half conus length. Procorpus cylindrical, narrowing at the junction with metacorpus. The latter, well-developed, muscular, longer than wide, 22 (20–25)×13 (12–15) μm sized, the granular part occupying 40 (35–45)% of its anterior part, the metacorpal valve plates slightly posterior to its middle, their mid-point 89 (81–96) μm from anterior end, well sclerotized. The junction of metacorpus with the intestine less than one metacarpal valve behind the metacorpus. Dorsal pharyngeal gland orifice opening at about one metacorpal valve length anterior to valve. Pharyngeal glands well developed, not well discernible from each other, overlapping intestine dorsally for 166 (145–186) μm, the glands end to anterior end 267 (244–283) μm. One of the pharyngeal glands’ nuclei usually visible, the other nuclei usually hardly visible due to granular texture of the glands. Intestine simple, ending in a blind sac, rectum not seen, anus vestigial in some specimens, lacking in most examined females. Excretory pore slightly more than one metacorpus length posterior to it. Hemizonid just posterior to excretory pore. Reproductive system monodelphic-prodelphic, occupying 49 (42.4–57.2)% of body (excluding postvulval uterine sac: PUS), composed of a single and outstretched ovary with oocytes mostly in multiple rows, oviduct variably long, spermatheca elongate, containing small amoeboid sperm in some individuals, crustaformeria-uterus junction not well discernible, vagina moderately sclerotized and straight, vulva a transverse slit, with a small epiptygma-like differentiation in lateral view, PUS tubular, its lumen distinct, lacking specific differentiation in junction with the uterus. Posterior body end (tail) conical, narrowing in distal end, ventrally bent, its tip finely rounded.

#### Male

Common in population, less than females, and seemingly functional (sperm observed inside the reproductive system of three females). Similar to female in general morphology, no sexual dimorphism in anterior region, posterior body end more strongly ventrally bent compared to posterior body end of the female. Reproductive system monorchic, occupying 30% of total body (T, n = 1), mature sperm observed in *vas deferens*. The latter part tube-like in posterior region, in distal end merges with distal end of intestine to form a simple tube connecting claoca. Spicules aphelenchoidid-type, 13±1 (12–13) μm long at mid-arc line, its dorsal arc-line 19±1 (19–20) μm, capitulum (condylus to rostrum distance) 9.5±0.5 (9–10) μm wide, condylus large, wide, blunt at the tip, rostrum small, pointed to blunt, the blade i.e. lamina-calomus complex ventrally arcuate, smoothly narrowing toward distal tip, the lamina more convex at middle, the tip bent dorsally. The single precloacal supplement (P1) lacking, the first precloacal papillae (P2) at 4.3 (3.0–5.5) μm distance anterior to cloacal opening, and the second pair of caudal papillae (P3) at 10.5 (8.5–13.0) μm distance to tail tip, both papillae mammiform and visible. Tail conical, ventrally bent, its tip sharp or blunt.

### Relationships

The new species was morphologically compared with species of the genus having three lines in the lateral field, similar posterior body end morphology and close morphometric data ranges as below (for the compared species lacking high quality light microphotograps, the comparison of stylet structure was not included):

Compared to *E*. *joyceae* Kaisa, Harman & Harman, 1995 [23], the new species has longer body (863.5 (772–926) *vs*. 640 (550–740) μm), longer stylet (23.8 (21.2–27.0) *vs*. 16 (14–18) μm) and different spicule morphology (tip dorsally bent *vs*. more or less straight).
Compared to *E*. *prolobos* Massey, 1964 [24], by lacking stylet knobs (*vs*. presence), having four precloacal and caudal papillae (*vs*. more than four; however, the exact number is not clear due to poor description), and basic difference is spicules morphology (spicules with dorsally bent tip *vs*. not).
Compared to *E*. *propora* (Yang, 1985 [25]) Ebsary, 1991 [26], a poorly described species, by shorter female body (863.5 (772–926) *vs*. 1083 (900–1260) μm, distinctly offset lip region separated from the rest body by a sharp constriction (*vs*. rounded, continuous with body, according to the drawing), and basic difference in spicules morphology (spicules with dorsally bent tip *vs*. not).
Compared to *E*. *riograndensis* Massey, 1964 [24], by greater a value (40.5 (35.9–45.2) *vs*. 25–34), greater b value (8.7 (8.2–9.5) *vs*. 6.4–8.5), narrow female posterior body end (*vs*. wide) and basic difference is spicules morphology (spicules with dorsally bent tip *vs*. not).
Compared to *E*. *taiwanensis*, the new species has basic difference in stylet characters [longer (23.8 (21.2–27.0) *vs*. 14.5 (12.0–16.9) μm), tripartite (*vs*. common-type) and well developed (*vs*. weaker)], longer PUS (20.8 (19–23) *vs*. 11.3 (8.8–15.3) μm) and spicules with a distally bent tip (*vs*. simple).
Compared to the population reported by Hunt and Hague [27] as *E*. *scolyti* Rühm, 1956 [28] (see Discussion), a species having similar spicules, the new species has three lines in the lateral field (*vs*. obscure, see Kaisa [29]), longer stylet (23.8 (21.2–27.0) *vs*. 17.3 (16–20) μm) and male with two pairs of precloacal and caudal papillae (*vs*. three).

### Iranian population of *Devibursaphelenchus teratospicularis* Kakulia & Devdariani, 1965

#### Measurements, morphology and distribution

**Fig 3:**
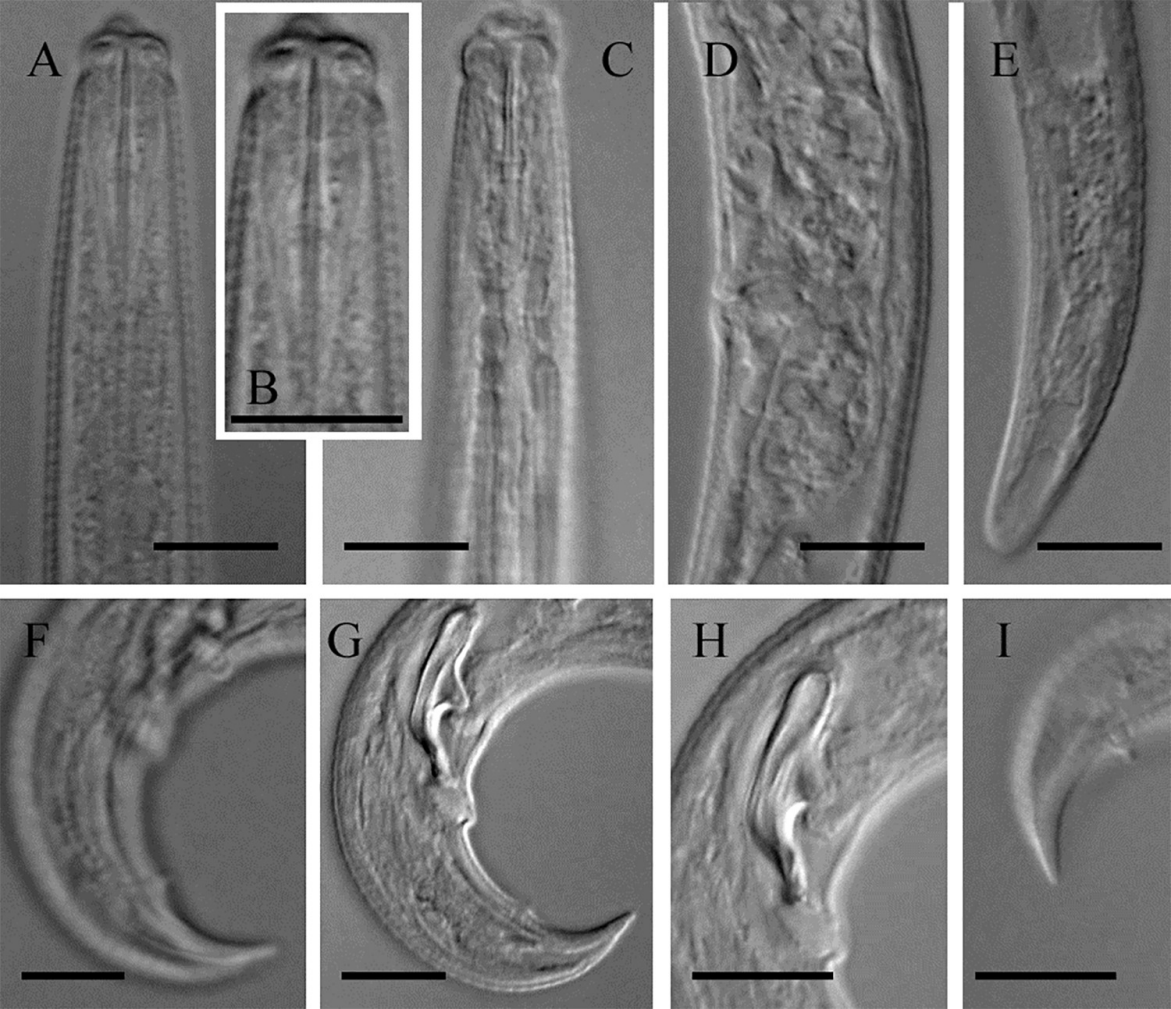
Iranian population of *Devibursaphelenchus teratospicularis* Kakulia & Devdariani, 1965 [13]. A-C: Anterior region of body (B: Stylet in higher magnitude), D: Vulva region and postvulval uterine sac (PUS), E: Female posterior body end, F&G: Male posterior body end, H: Spicules, I: Bursa in lateral view. (All scale bars = 10 μm).

Morphometric variability is described in Table 1 and morphological traits shown in Fig 3.

### Habitat and locality

The Iranian population of *D*. *teratospicularis* was recovered from bark samples of dead *Pinus* sp. having frass and galleries of bark beetles, collected in Chitgar forest park, city of Tehran, Tehran province, Iran, collected on late 2016-early 2017.

#### Female

Body slender, slightly ventrally curved after fixation, very gradually narrowing toward both ends. Cuticle distinctly annulated, annulus ca. 2 μm wide. Lateral field with three lines. Lip region separated from the rest of the body by a distinct constriction, 2.1 (2.0–2.3) times wider than tall, flat at anterior end, lips separated, their size not well discernible in lateral view. Stylet well developed with wide lumen, tripartite, composed of a conus with an aperture about half its length, a conophore slightly shorter than the conus, and a shaft, around twice conus length long, lacking basal knobs or swellings. The stylet has a well visible lumen throughout the stylet. Procorpus boundaries not clearly seen, metacorpus well-developed, muscular, longer than wide, 19.5 (18–20)×11 (10–12) μm sized, the granular part occupying 41 (40–45)% of its anterior part, the metacorpal valve plates located slightly posterior to middle of metacorpus, their mid-point 73 (66–78) μm from anterior end, well sclerotized, its junction with the intestine just behind the metacorpus. Dorsal pharyngeal gland orifice at slightly less than one metacorpal valve length anterior to the valve. Pharyngeal glands well developed, their border not discernible, overlapping intestine dorsally for 158 (128–174) μm, anterior end to end of glands 235 (205–251) μm. Intestine simple, ending in a blind sac, rectum not seen, anus vestigial. Excretory pore at more than one metacorpus length posterior to it. Hemizonid just posterior to excretory pore (n = 1). Reproductive system monodelphic-prodelphic, ovary outstretched, the oocytes mostly in multiple rows, oviduct variably long, spermatheca elongate, containing small amoeboid sperm, crustaformeria-uterus junction not well discernible, vagina moderately sclerotized, ca. 44% corresponding body width in length, straight, vulva a transverse slit, PUS tubular, 1.1 (0.8–1.3) times vulval body width long, apparently lacking specific differentiation in junction with the uterus. Posterior body end (tail) conical, with widely rounded tip.

#### Male

Rare (only one male was recovered). Similar to female in general morphology, except for reproductive system and posterior body end more strongly ventrally bent. Reproductive system monorchic, occupying 47% of total body (T). Spicules 13 μm long at mid-arc line, its dorsal arc-line 21 μm, capitulum (condylus to rostrum) 10 μm wide, the condylus large, having widely rounded end, rostrum small, blunt and wide, spicules tip with a small apophysis, bent ventrally. The single precloacal supplement (P1) lacking, the first precloacal papillae (P2) at 9 μm distance anterior to cloacal opening, and the second pair of caudal papillae (P3) at 14 μm distance to tail tip, both papillae mammiform and visible. Tail conical, ventrally bent. The bursa well visible in lateral view, sharp.

### Remarks

No remarkable differences in morphometric data were observed between the Iranian and the type population. The original drawing of the species is also poor, and some features like stylet status is not well drawn. A functional or vestigial anus is however not illustrated, while the index "c" is given for the female [13]. Again, a functional anus and rectum is drawn for the species by Kakulia [30], showing the studied population might probably have had a vestigial anus. Females of the presently studied Iranian population had a vestigial anus. The species is reported from Russia and some factors related with its spread are also studied [31]. No morphological or morphometric data are given for this Russian population. In 2006, the species was found in Portugal in association with *Orthotomicus erosus* Wollaston, 1857 [32], where the nematode was discovered inside the body of the insect [33]. Surprisingly, the Iranian population of *D*. *teratospicularis* was also recovered from wood and bark samples of dead *Pinus* sp. trees collected from the Chitgar forest park in city of Tehran, that were heavily infected with the aforementioned beetle. Based on the light microphotograps given for the Portuguese population (including male and female posterior body end characters, spicules morphology, cuticle characters, and PUS characters), no remarkable differences are seen between this population and the Iranian population. Individuals of the Iranian population were not recovered from inside the body of the seven dissected beetles. However, a repeating of this experiment is needed, as, only few individuals of beetles were examined. The species was reported from Ukraine by Gu et al. [34]; and the light microphotographs, morphometric and molecular data (sequences of SSU and LSU rDNA D2-D3) were made available for it. Compared to this population, again, no remarkable morphological and morphometric differences were observed (for molecular comparisons, see below).

### Molecular phylogenetic analyses

For molecular phylogenetic studies, two isolates of *Devibursaphelenchus teratospicularis* and three isolates of the new species, *Ektaphelenchus kanzakii* n. sp. were used. One partial SSU sequence was amplified and sequenced for each species (the accession number MH595681 for *Devibursaphelenchus teratospicularis*; and MH595682 for the new species). Five sequences were obtained for LSU D2-D3 fragments (MH595683 and MH595684 for two isolates of *Devinbrsaphelenchus teratospicularis*; and MH595685 and MH595686 for two isolates of the new species).

The BLAST search using the partial SSU of *Devinbrsaphelenchus teratospicularis* revealed it had 99% identity with the SSU sequences of this species from Ukraine (KC148531) (20 indels, 7 gaps). The BLAST search using SSU rDNA of the new species revealed it had 99% identity (6 indels, 3 gaps) with *Ektaphelenchus obtusus* Massey, 1956 [35] (AB368532). The BLAST search using the partial LSU D2-D3 sequences of *Devibursaphelenchus teratospicularis* (MH595683 and MH595684, identical) revealed they had 98% identity with *D*. *teratospicularis* from Ukraine (KC148532) (16 indels, 1 gap). The BLAST search using the partial LSU D2-D3 sequences of *Ektaphelenchus kanzakii* n. sp. (MH595685 and MH595686, identical) revealed they had at maximum 92% identity with currently available sequences deposited into the GenBank database (*E*. *obtusus*, AB368533; further than 40 indels and 10 gaps). The identity value with all other sequences were less than 92%.

**Fig 4:**
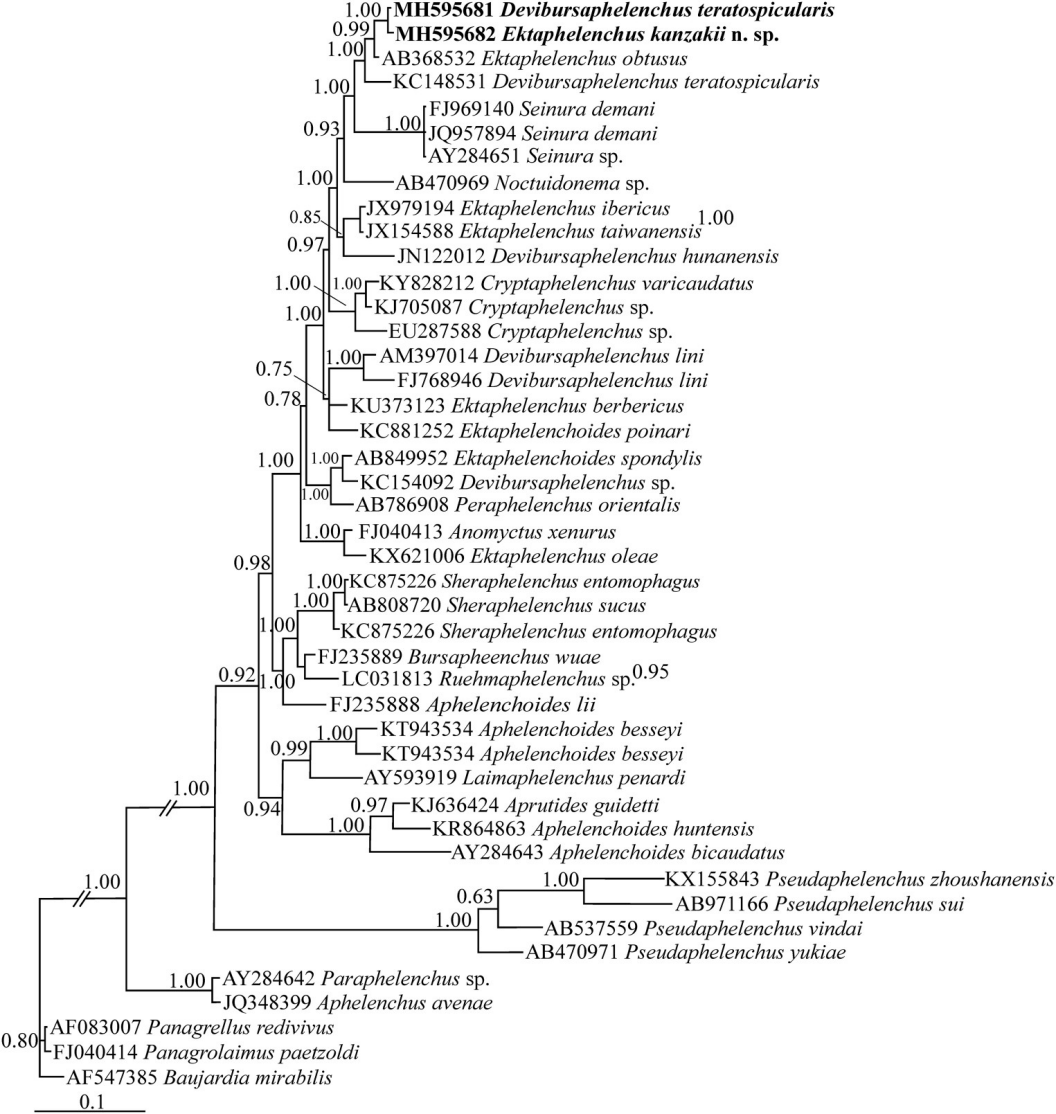
Bayesian 50% majority rule consensus tree inferred from SSU rDNA of *Ektaphelenchus kanzakii* n. sp. and Iranian population of *Devibursaphelenchus teratospicularis* Kakulia & Devdariani, 1965 [13] under the GTR + G + I model. Bayesian posterior probabilities (BPP) more than 50% are given for appropriate clades. New sequences are in bold font.

For phylogenetic analyses, two separate SSU and LSU datasets were prepared. The SSU dataset was composed of 44 species/isolates (including three bacterivorous rhabditids as outgroup taxa). The dataset had 1436 total characters of which 720 characters were variable. In this tree (Fig 4), two presently studied Iranian ektaphelenchid species have formed a maximally supported clade (Bayesian posterior probability: BPP = 1.00) and the clade including the new species, the Iranian and Ukrainian isolates of *Devibursaphelenchus teratospicularis* and *Ektaphelencus obtusus* has received the maximal BPP.

**Fig 5:**
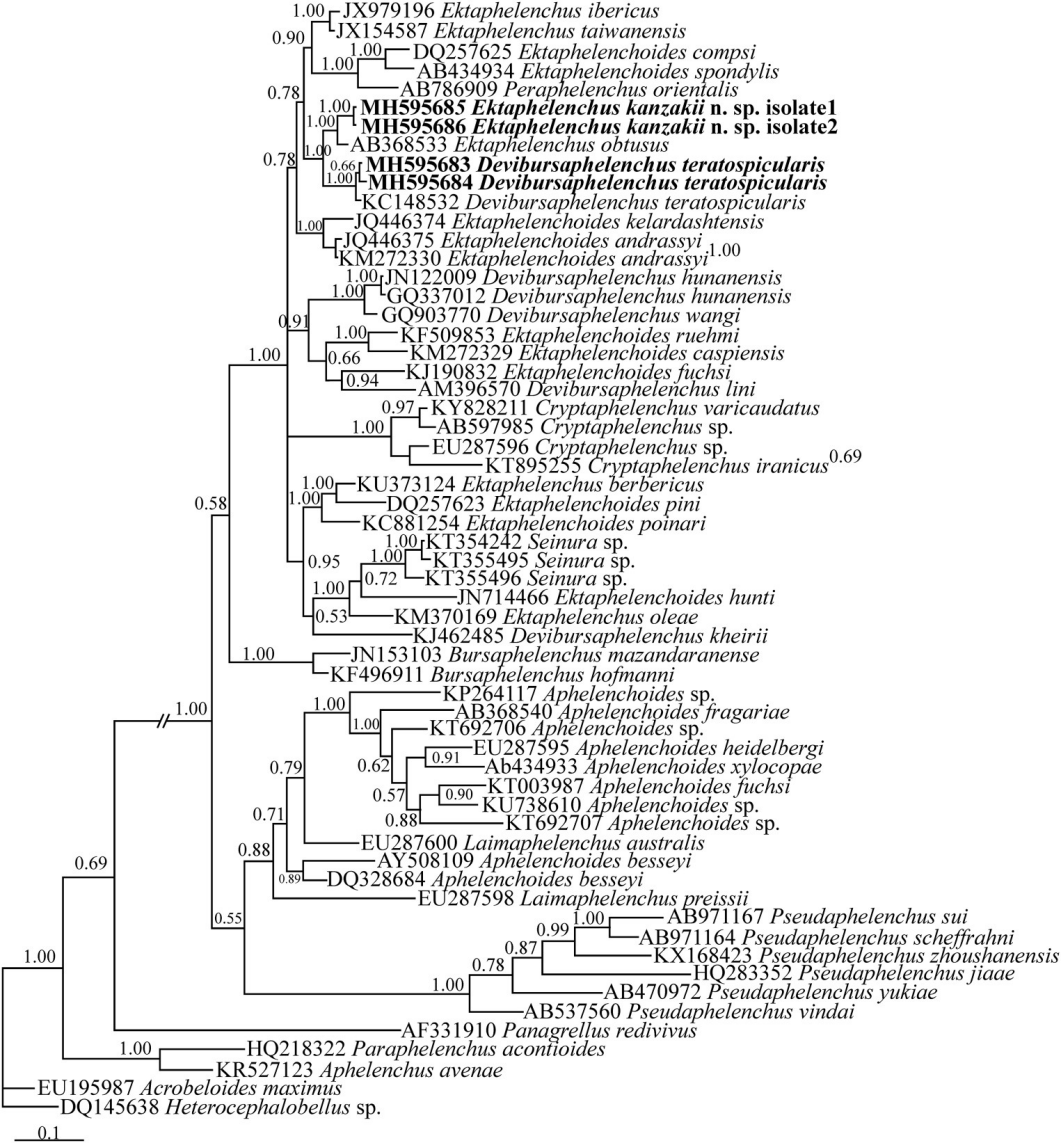
Bayesian 50% majority rule consensus tree inferred from LSU rDNA D2-D3 of *Ektaphelenchus kanzakii* n. sp. and Iranian population of *Devibursaphelenchus teratospicularis* Kakulia & Devdariani, 1965 [13] under the GTR + G + I model. Bayesian posterior probabilities (BPP) more than 50% are given for appropriate clades. New sequences are in bold font.

The LSU dataset was composed of 59 species/isolates (including two bacterivorous rhabditids as outgroup taxa). The dataset had 498 total characters of which 309 characters were variable. In this tree (Fig 5), the new species has formed a maximally supported clade with *Ektaphelenchus obtusus* and the Iranian and the Ukrainian isolates of *Devibursaphelenchus teratospicularis* have formed a maximally supported clade. The clade including three aforementioned species (*Ektaphelenchus obtusus*, *E*. *kanzakii* n. sp. and *Devibursaphelenchus teratospicularis*) is maximally supported (BPP = 1.00).

## Discussion

The continued studies on identification of ektaphelenchid nematodes occurring in Iran, yielded two species illustrated and studied in present paper. The new species *Ektaphelenchus kanzakii* n. sp. was described based upon morphological and molecular data. It has a three partite stylet, as described, and an epiptygma-like projection in the vulva. A new term was proposed for the middle part of the stylet, the conophore, differentiating the ektaphelenchid-type tripartite stylet from the common tripartite stylet seen in most non-ektaphelenchid tylenchs (which have conus + shaft + knobs).

The same three partite stylet structure was also observed for the recovered population of *Devibursaphelenchus teratospicularis*. A close inspecting of available light microphotographs of the species *Ektaphelenchus oleae* (Fig 3A, original description), *E*. *berbericus* (Fig 2B, original description), *E*. *obtusus* (Figs 9C and 11A within [36]) and *Devibursaphelenchus teratospicularis* (Fig 1D within [34]), revealed the aforementioned species/populations also have the same stylet structure, i.e. the stylet is composed of a conus, a conophore and a shaft. This type of stylet was already reported for *Ektaphelenchoides spondylis* Kanzaki, Giblin-Davis & Center 2009 [37] and is apparently seen in light microphotographs of *E*. *poinari* Aliramaji, Pourjam, Atighi, Ye, Roshan-Bakhsh & Pedram, 2014 [38] too (Fig 2B, original description). The high quality light microphotographs were not available for most ektaphelenchids, and it seems this type of stylet could be common for the genera *Ektaphelenchus*, *Ektaphelenchoides* Baujard, 1984 [39] and *Devibursaphelenchus*.

*Seinura musicola* (Timm, 1960 [40]) Andrássy, 2007 [41] also has a stylet of this type, and this shared feature, emphasizes on further studies on taxonomy of ektaphelenchids and seinurids. The two close genera *Ektaphelenchoides* and *Seinura* Fuchs, 1931 [42] share many morphological traits and are separated from each other by the status of rectum and anus (functional in *Seinura vs*. absent or vestigial in *Ektaphelenchoides*) (see below). The two presently studied ektaphelenchid species had simple stylet base, and no swellings in any form (knob-like or club-like base, or gradually widening status) was observed for them.

In both SSU and LSU phylogenies discussed in present study, the nonmonophyletic nature of three genera *Ektaphelenchus*, *Ektaphelenchoides* and *Devibursaphelenchus* is seen (polyphyletic nature of *Ektaphelenchus* and *Devinursaphelenchus* and paraphyletic nature (this could be due to only two available sequences) of *Ektaphelenchoides* in SSU tree; and polyphyletic nature of all three genera in LUS tree) that is in congruence with molecular phylogenetic studies of these three genera in the last years. It is however surprising that *Cryptaphelenchus* with currently sequenced species is monophyletic in both SSU and LSU phylogenies. This status could further corroborate usefulness of SSU and LSU data for direct generic identification of the latter genus. The subfamily, by itself, is again nonmonophyletic and species of *Noctuidonema* Remillet & Silvain, 1988 [43], *Peraphelenchus* Wachek, 1955 [44] and *Anomyctus* Allen, 1940 [45] occupy placements inside this subfamily in both trees. The ongoing molecular phylogenetic studies on the subfamily are however promising and could yield on more reliable trees in future, as molecular data are accumulating, although slowly. A future phylogenetic study on the subfamily will need further data of several representatives, and would also need further data from several real *Seinura* spp. to be included. Using other genomic or non-genomic markers could be the alternative option, still needs to be exploited.

Both of the studied species in present paper were recovered from wood and bark samples, that besides these two species, harbored a diplogasterid nematode and some tylenchids in high and low population, respectively. The biology of Ektaphelenchinae Paramonov, 1964 [46] is recently reviewed by Kanzaki and Giblin-Davis [47]. Based upon currently available data on predatory feeding habitat of both genera *Devibursaphelenchus* and *Ektaphelenchus* [3, 48], the predatory feeding habit is assumed for presently studied two species too, and the well-developed stylet apparatus of both species could support this hypothesis. It is however surprising that the subfamily has a wide range of feeding type. Besides their relation with insects, predatory and insect parasitism [47], the mycetophagous feeding habit is also documented for the genus *Cryptaphelenchus* [49] and again, it seems the stylet characters of this genus is well suitable for this purpose. These observations corroborate the subfamily has a wide range of feeding type.

In present study, I found the studied and illustrated population of *Ektaphelenchus* by Hunt and Hague [27] as *E*. *scolyti*, looks very similar to the population identified as *E*. *scolyti* by Kaisa [29]. It was surprising that besides general morphological and morphometric concordance, the spicules characters, i.e. having a dorsally bent apophasis in spicules distal end confirm these two populations could be conspecific. However, the population of Kaisa lacks stylet knobs, while, Hunt and Hague [27] have illustrated stylet knobs, and again, stated it lacks stylet knobs in description. The type population of *E*. *scolyti* on the other hand, is poorly illustrated, but, seems the species has small knobs (“stylet similar to that in *E*. *betulae* Rühm, 1956 [28]”, as stated by Rühm). The population of Hunt and Haque, however, has remarkable differences with the type population of *E*. *scolyti*. More remarkably, the V value of Hunt and Hague population (77.2 (76–79)) differs from the range given for the type population (65.5–75.5), and the spicules tip is dorsally bent (vs. simple in original drawing of *E*. *scolyti* by Rühm). As the result, the populations of Hunt and Hague [25] and Kaisa [29] could be conspecific, different from the type population of *E*. *scolyti*, and by having accessible type material, could be erected as an independent and possibly new species after a comparison with valid species of the genus, an action that is beyond the aims of the present study.

And finally, no high quality description and drawings were found for the species *E*. *chalcographi* Kurashvili, Kakulia & Devdariani, 1980 [50] while my morphological comparisons in present study. Its stylet length (9 μm) needs further confirmations, and the V value is not given for it [50]. Furthermore, no data was available for the type materials and as the result, this species is not valid.
